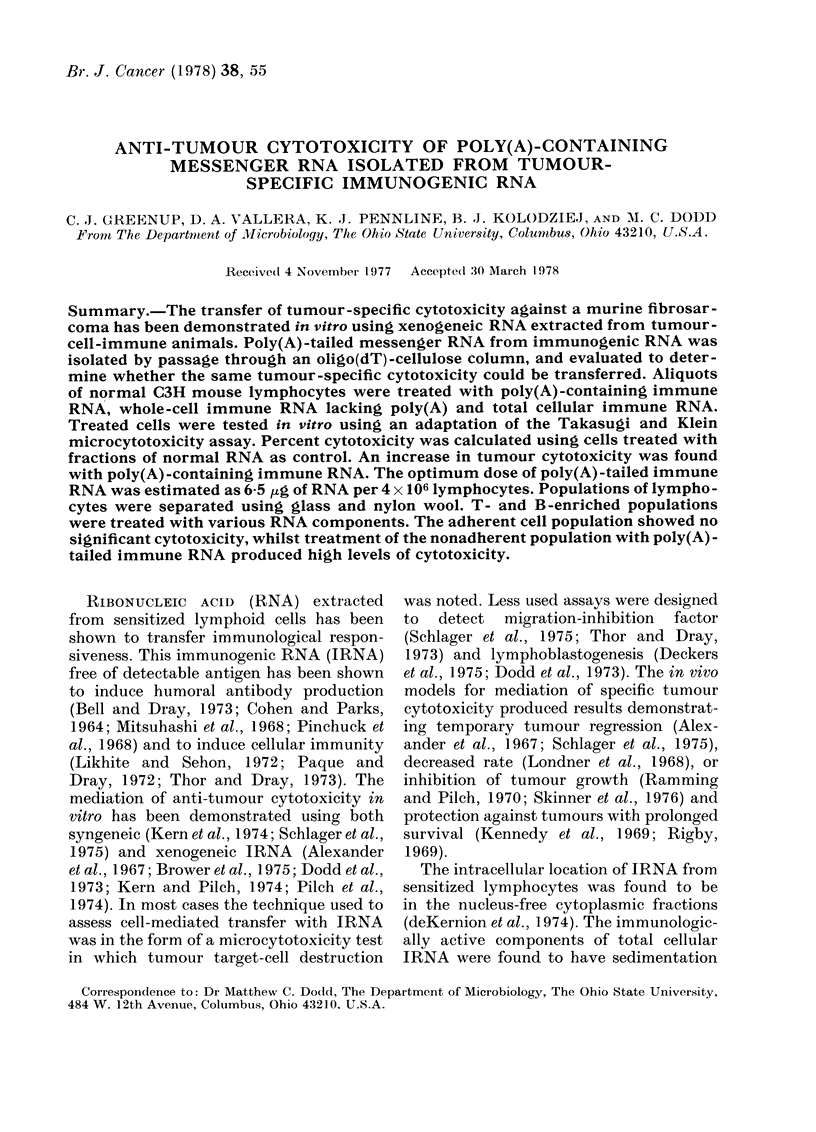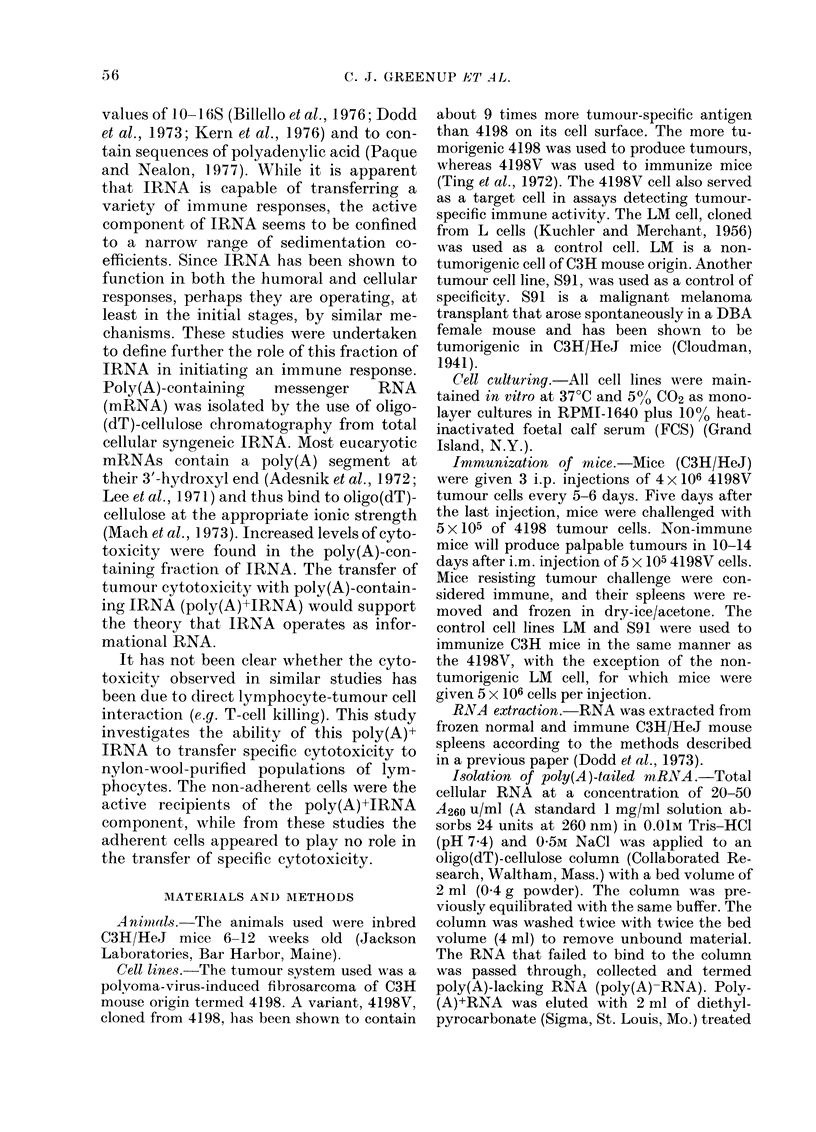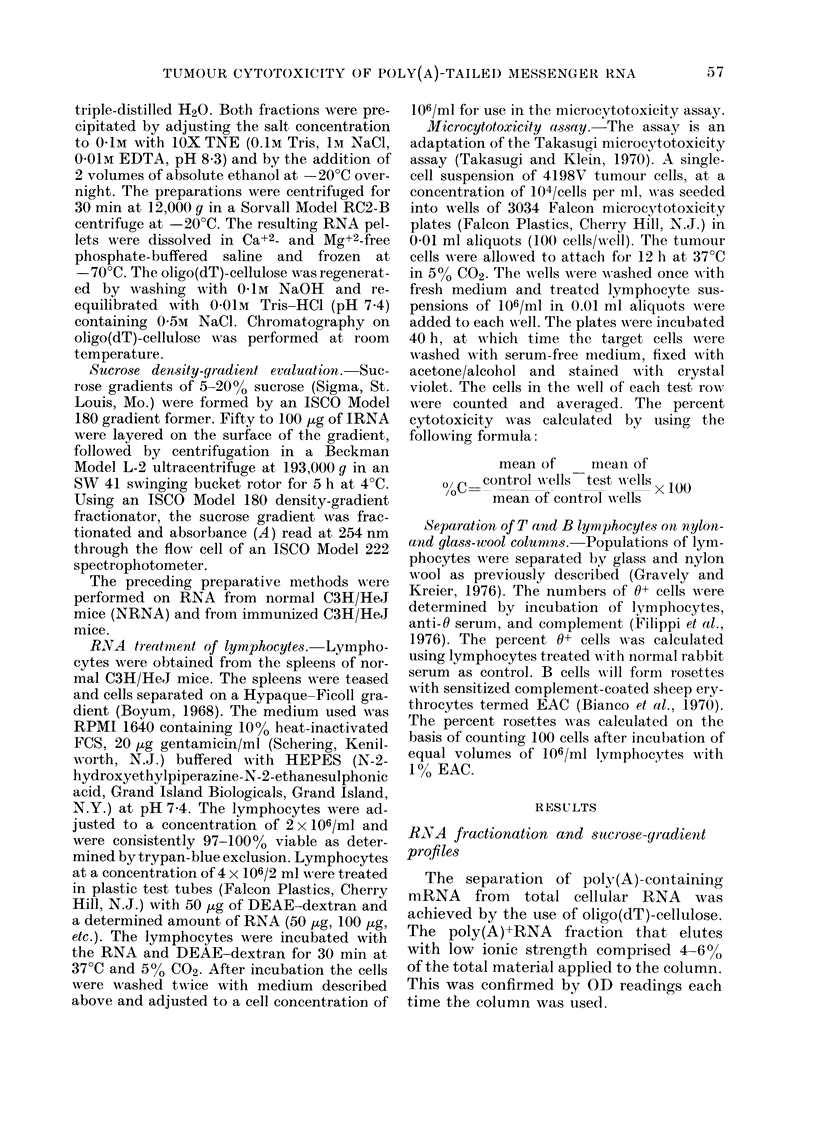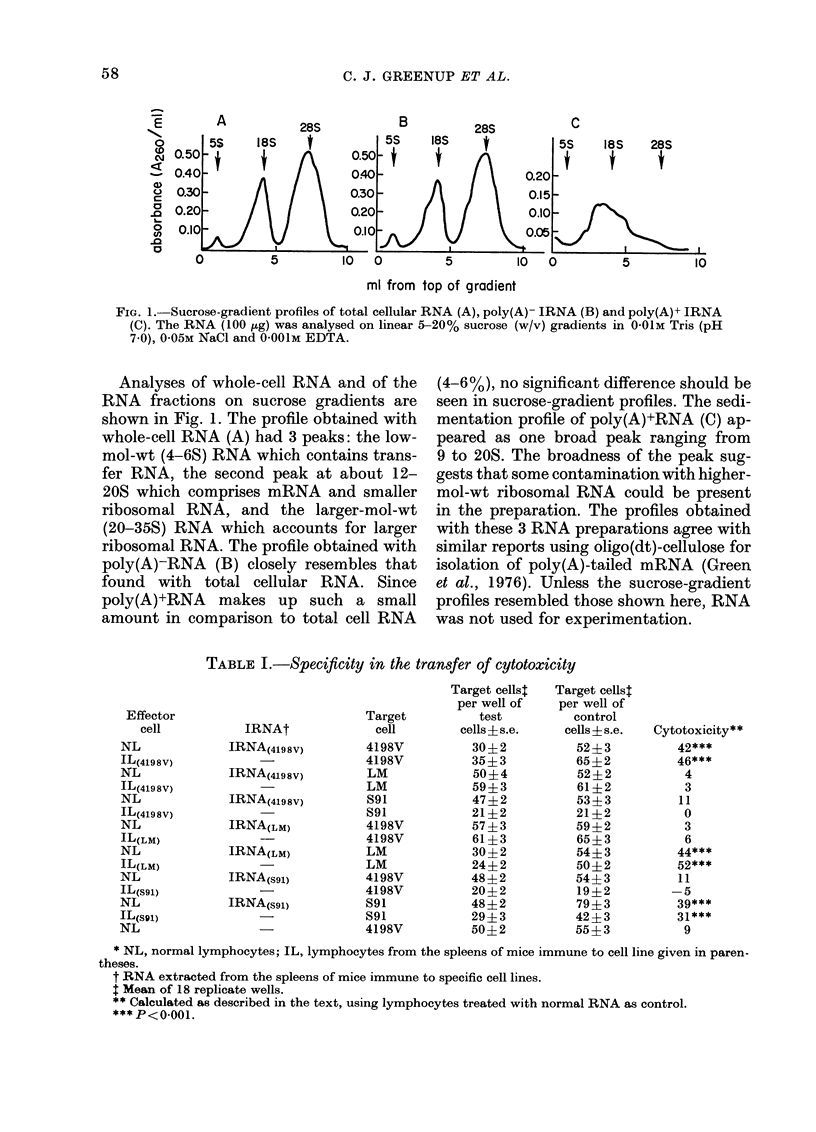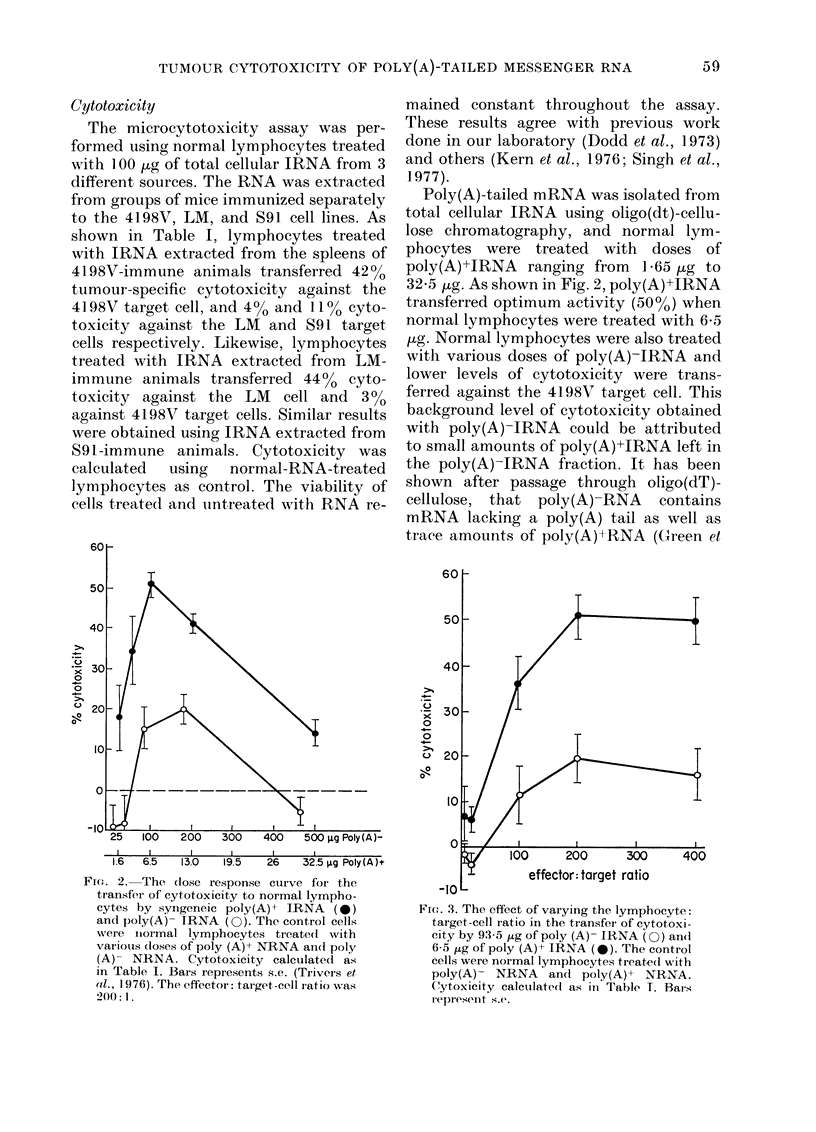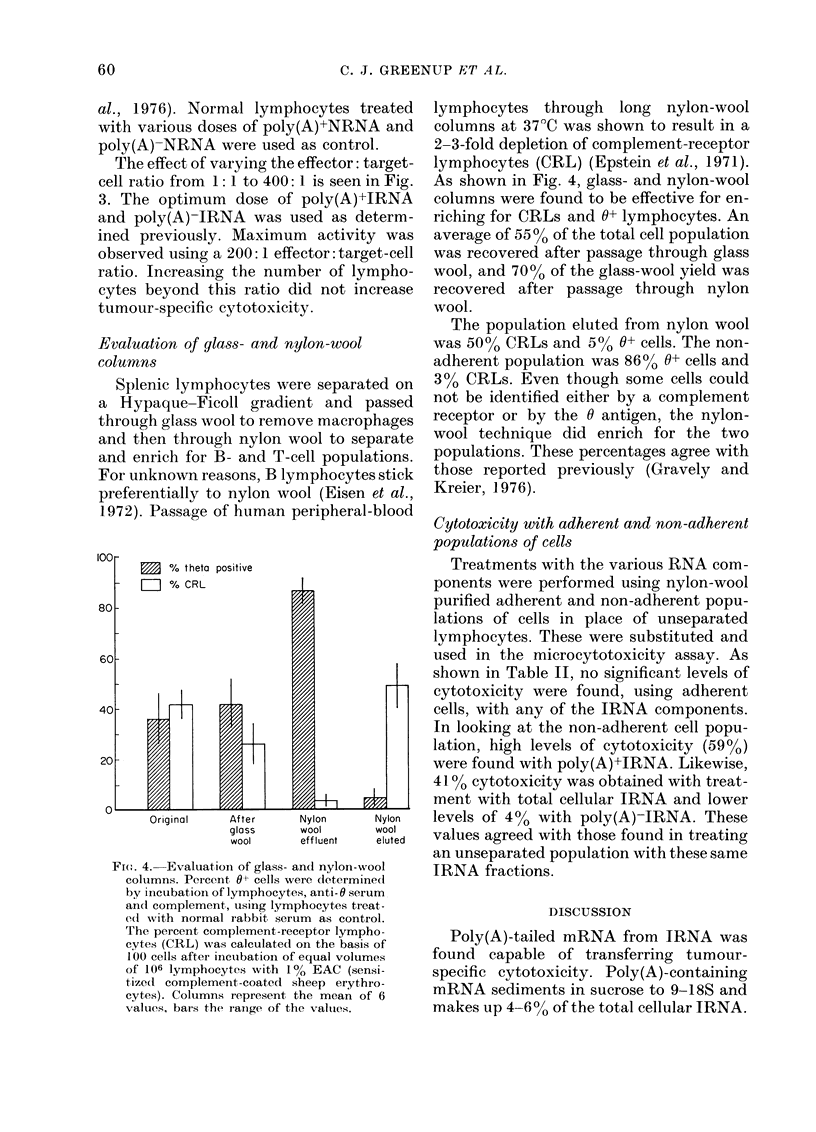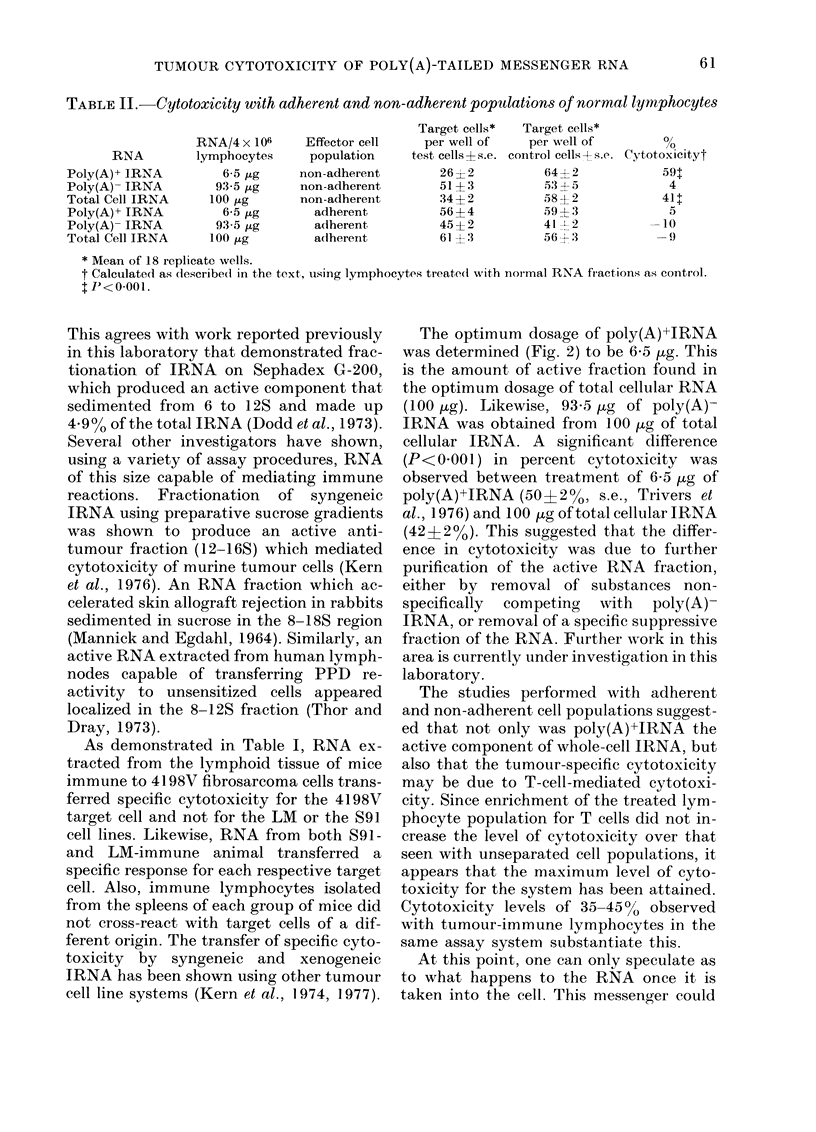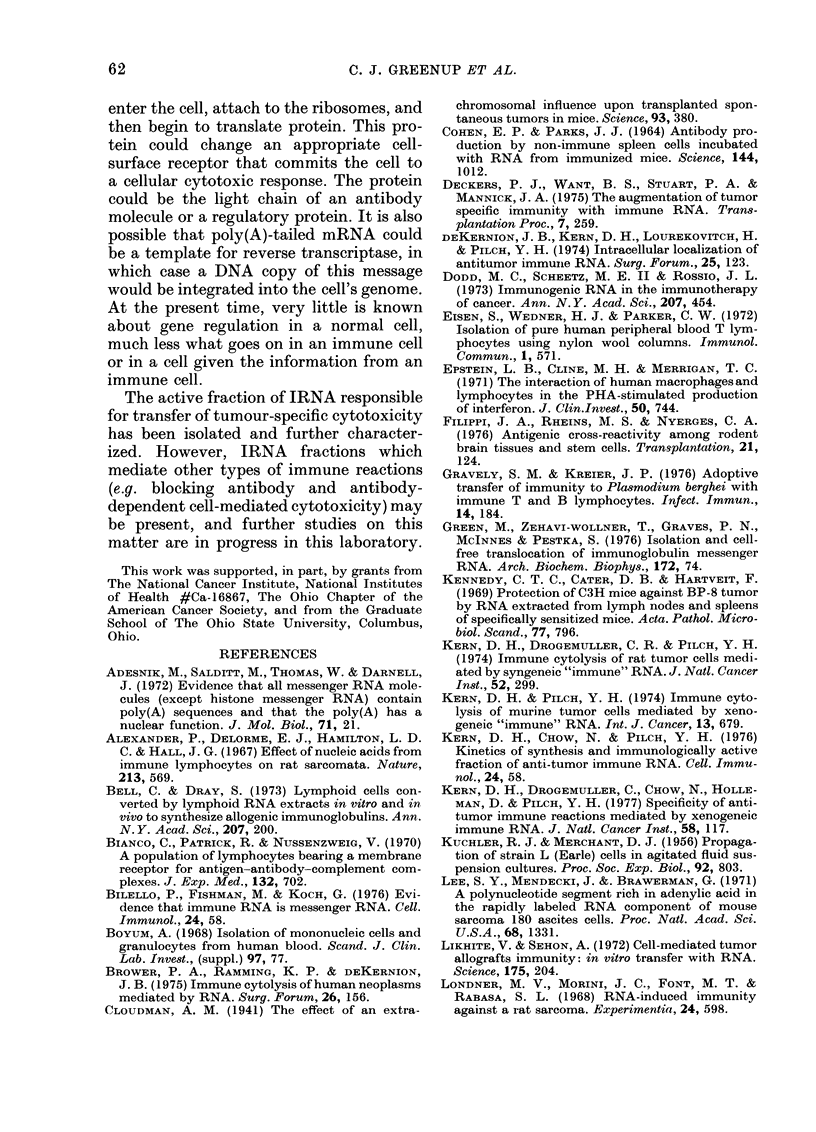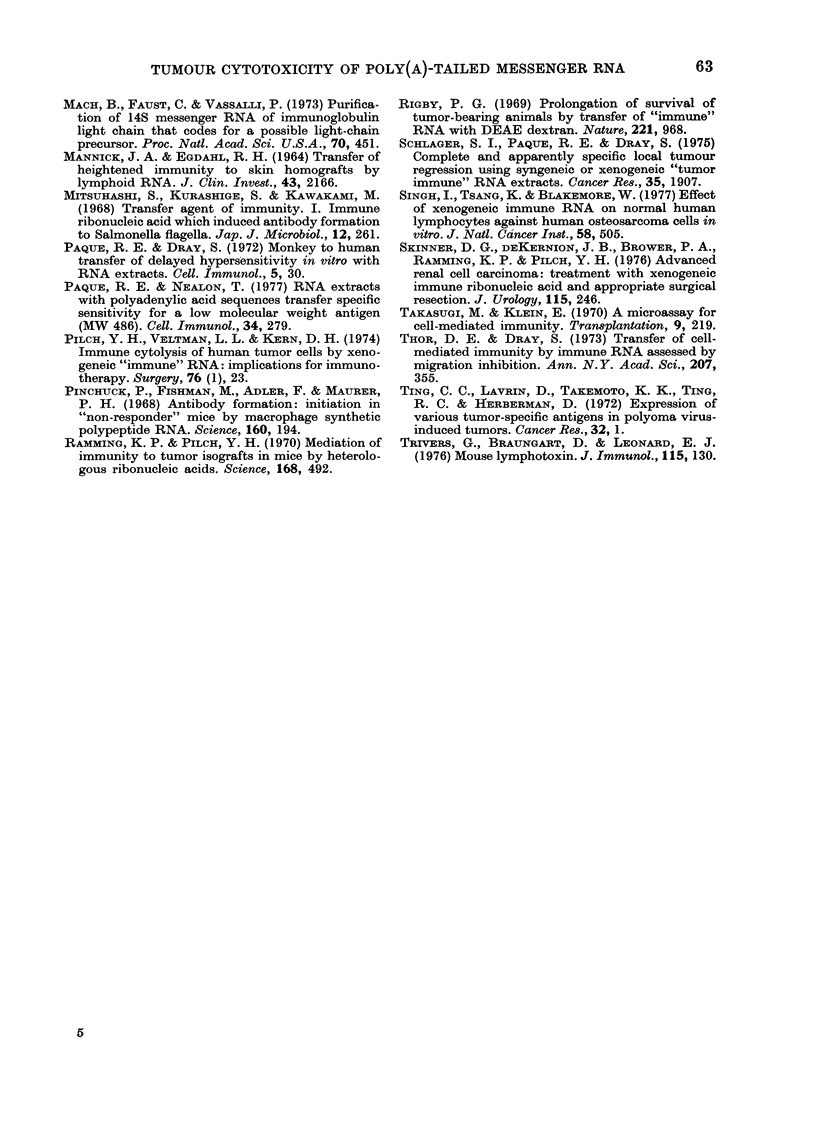# Anti-tumour cytotoxicity of poly(A)-containing messenger RNA isolated from tumour-specific immunogenic RNA.

**DOI:** 10.1038/bjc.1978.163

**Published:** 1978-07

**Authors:** C. J. Greenup, D. A. Vallera, K. J. Pennline, B. J. Kolodziej, M. C. Dodd

## Abstract

The transfer of tumour-specific cytotoxicity against a murine fibrosar-coma has been demonstrated in vitro using xenogeneic RNA extracted from tumour-cell-immune animals. Poly(A)-tailed messenger RNA from immunogenic RNA was isolated by passage through an oligo(dT)-cellulose column, and evaluated to determine whether the same tumour-specific cytotoxicity could be transferred. Aliquots of normal C3H mouse lymphocytes were treated with poly(A)-containing immune RNA, whole-cell immune RNA lacking poly(A) and total cellular immune RNA. Treated cells were tested in vitro using an adaptation of the Takasugi and Klein microcytotoxicity assay. Percent cytotoxicity was calculted using cells treated with fractions of normal RNA as control. An increase in tumour cytotoxicity was found with poly(A)-containing immune RNA. The optimum dose of poly(A)-tailed immune RNA was estimated as 6.5 microgram of RNA per 4 x 10(6) lymphocytes. Populations of lymphocytes were separated using glass and nylon wool. T- and B-enriched populations were treated with various RNA components. The adherent cell population showed no significant cytotoxicity, whilst treatment of the nonadherent population with poly(A)-tailed immune RNA produced high levels of cytotoxicity.


*Br. J. Cancer* (1978) **38**, 55

### ANTI-TUMOUR CYTOTOXICITY OF POLY(A)-CONTAINING MESSENGER RNA ISOLATED FROM TUMOUR-SPECIFIC IMMUNOGENIC RNA

C. J. GREENUP, D. A. VALLERA, K. J. PENNLINE, B. J. KOLODZIEJ, AND M. C. DODD
*From The Department of Microbiology, The Ohio State University, Columbus, Ohio 43210, U.S.A.*

Received 4 November 1977 Accepted 30 March 1978

**Summary.—The transfer of tumour-specific cytotoxicity against a murine fibrosarcoma has been demonstrated *in vitro* using xenogeneic RNA extracted from tumour-cell-immune animals. Poly(A)-tailed messenger RNA from immunogenic RNA was isolated by passage through an oligo(dT)-cellulose column, and evaluated to determine whether the same tumour-specific cytotoxicity could be transferred. Aliquots of normal C3H mouse lymphocytes were treated with poly(A)-containing immune RNA, whole-cell immune RNA lacking poly(A) and total cellular immune RNA. Treated cells were tested *in vitro* using an adaptation of the Takasugi and Klein microcytotoxicity assay. Percent cytotoxicity was calculated using cells treated with fractions of normal RNA as control. An increase in tumour cytotoxicity was found with poly(A)-containing immune RNA. The optimum dose of poly(A)-tailed immune RNA was estimated as 6·5 $\mu$g of RNA per $4 \times 10^6$ lymphocytes. Populations of lymphocytes were separated using glass and nylon wool. T- and B-enriched populations were treated with various RNA components. The adherent cell population showed no significant cytotoxicity, whilst treatment of the nonadherent population with poly(A)-tailed immune RNA produced high levels of cytotoxicity.**

Ribonucleic acid (RNA) extracted from sensitized lymphoid cells has been shown to transfer immunological responsiveness. This immunogenic RNA (IRNA) free of detectable antigen has been shown to induce humoral antibody production (Bell and Dray, 1973; Cohen and Parks, 1964; Mitsuhashi *et al.*, 1968; Pinchuck *et al.*, 1968) and to induce cellular immunity (Likhite and Sehon, 1972; Paque and Dray, 1972; Thor and Dray, 1973). The mediation of anti-tumour cytotoxicity *in vitro* has been demonstrated using both syngeneic (Kern *et al.*, 1974; Schlager *et al.*, 1975) and xenogeneic IRNA (Alexander *et al.*, 1967; Brower *et al.*, 1975; Dodd *et al.*, 1973; Kern and Pilch, 1974; Pilch *et al.*, 1974). In most cases the technique used to assess cell-mediated transfer with IRNA was in the form of a microcytotoxicity test in which tumour target-cell destruction was noted. Less used assays were designed to detect migration-inhibition factor (Schlager *et al.*, 1975; Thor and Dray, 1973) and lymphoblastogenesis (Deckers *et al.*, 1975; Dodd *et al.*, 1973). The *in vivo* models for mediation of specific tumour cytotoxicity produced results demonstrating temporary tumour regression (Alexander *et al.*, 1967; Schlager *et al.*, 1975), decreased rate (Londner *et al.*, 1968), or inhibition of tumour growth (Ramming and Pilch, 1970; Skinner *et al.*, 1976) and protection against tumours with prolonged survival (Kennedy *et al.*, 1969; Rigby, 1969).

The intracellular location of IRNA from sensitized lymphocytes was found to be in the nucleus-free cytoplasmic fractions (deKernion *et al.*, 1974). The immunologically active components of total cellular IRNA were found to have sedimentation

Correspondence to: Dr Matthew C. Dodd, The Department of Microbiology, The Ohio State University, 484 W. 12th Avenue, Columbus, Ohio 43210, U.S.A.

values of 10–16S (Billello *et al.*, 1976; Dodd *et al.*, 1973; Kern *et al.*, 1976) and to contain sequences of polyadenylic acid (Paque and Nealon, 1977). While it is apparent that IRNA is capable of transferring a variety of immune responses, the active component of IRNA seems to be confined to a narrow range of sedimentation coefficients. Since IRNA has been shown to function in both the humoral and cellular responses, perhaps they are operating, at least in the initial stages, by similar mechanisms. These studies were undertaken to define further the role of this fraction of IRNA in initiating an immune response. Poly(A)-containing messenger RNA (mRNA) was isolated by the use of oligo-(dT)-cellulose chromatography from total cellular syngeneic IRNA. Most eucaryotic mRNAs contain a poly(A) segment at their 3′-hydroxyl end (Adesnik *et al.*, 1972; Lee *et al.*, 1971) and thus bind to oligo(dT)-cellulose at the appropriate ionic strength (Mach *et al.*, 1973). Increased levels of cytotoxicity were found in the poly(A)-containing fraction of IRNA. The transfer of tumour cytotoxicity with poly(A)-containing IRNA (poly(A)$^+$IRNA) would support the theory that IRNA operates as informational RNA.

It has not been clear whether the cytotoxicity observed in similar studies has been due to direct lymphocyte-tumour cell interaction (*e.g.* T-cell killing). This study investigates the ability of this poly(A)$^+$ IRNA to transfer specific cytotoxicity to nylon-wool-purified populations of lymphocytes. The non-adherent cells were the active recipients of the poly(A)$^+$IRNA component, while from these studies the adherent cells appeared to play no role in the transfer of specific cytotoxicity.

### MATERIALS AND METHODS

*Animals.*—The animals used were inbred C3H/HeJ mice 6–12 weeks old (Jackson Laboratories, Bar Harbor, Maine).

*Cell lines.*—The tumour system used was a polyoma-virus-induced fibrosarcoma of C3H mouse origin termed 4198. A variant, 4198V, cloned from 4198, has been shown to contain about 9 times more tumour-specific antigen than 4198 on its cell surface. The more tumorigenic 4198 was used to produce tumours, whereas 4198V was used to immunize mice (Ting *et al.*, 1972). The 4198V cell also served as a target cell in assays detecting tumour-specific immune activity. The LM cell, cloned from L cells (Kuchler and Merchant, 1956) was used as a control cell. LM is a non-tumorigenic cell of C3H mouse origin. Another tumour cell line, S91, was used as a control of specificity. S91 is a malignant melanoma transplant that arose spontaneously in a DBA female mouse and has been shown to be tumorigenic in C3H/HeJ mice (Cloudman, 1941).

*Cell culturing.*—All cell lines were maintained *in vitro* at 37°C and 5% $CO_2$ as monolayer cultures in RPMI-1640 plus 10% heat-inactivated foetal calf serum (FCS) (Grand Island, N.Y.).

*Immunization of mice.*—Mice (C3H/HeJ) were given 3 i.p. injections of $4 \times 10^6$ 4198V tumour cells every 5–6 days. Five days after the last injection, mice were challenged with $5 \times 10^5$ of 4198 tumour cells. Non-immune mice will produce palpable tumours in 10–14 days after i.m. injection of $5 \times 10^5$ 4198V cells. Mice resisting tumour challenge were considered immune, and their spleens were removed and frozen in dry-ice/acetone. The control cell lines LM and S91 were used to immunize C3H mice in the same manner as the 4198V, with the exception of the non-tumorigenic LM cell, for which mice were given $5 \times 10^6$ cells per injection.

*RNA extraction.*—RNA was extracted from frozen normal and immune C3H/HeJ mouse spleens according to the methods described in a previous paper (Dodd *et al.*, 1973).

*Isolation of poly(A)-tailed mRNA.*—Total cellular RNA at a concentration of 20–50 $A_{260}$ u/ml (A standard 1 mg/ml solution absorbs 24 units at 260 nm) in 0.01M Tris–HCl (pH 7·4) and 0·5M NaCl was applied to an oligo(dT)-cellulose column (Collaborated Research, Waltham, Mass.) with a bed volume of 2 ml (0·4 g powder). The column was previously equilibrated with the same buffer. The column was washed twice with twice the bed volume (4 ml) to remove unbound material. The RNA that failed to bind to the column was passed through, collected and termed poly(A)-lacking RNA (poly(A)$^-$RNA). Poly-(A)$^+$RNA was eluted with 2 ml of diethylpyrocarbonate (Sigma, St. Louis, Mo.) treated triple-distilled $H_2O$. Both fractions were precipitated by adjusting the salt concentration to 0·1M with 10X TNE (0.1M Tris, 1M NaCl, 0·01M EDTA, pH 8·3) and by the addition of 2 volumes of absolute ethanol at −20°C overnight. The preparations were centrifuged for 30 min at 12,000 *g* in a Sorvall Model RC2-B centrifuge at −20°C. The resulting RNA pellets were dissolved in $Ca^{+2}$- and $Mg^{+2}$-free phosphate-buffered saline and frozen at −70°C. The oligo(dT)-cellulose was regenerated by washing with 0·1M NaOH and reequilibrated with 0·01M Tris–HCl (pH 7·4) containing 0·5M NaCl. Chromatography on oligo(dT)-cellulose was performed at room temperature.

*Sucrose density-gradient evaluation.*—Sucrose gradients of 5–20% sucrose (Sigma, St. Louis, Mo.) were formed by an ISCO Model 180 gradient former. Fifty to 100 μg of IRNA were layered on the surface of the gradient, followed by centrifugation in a Beckman Model L-2 ultracentrifuge at 193,000 *g* in an SW 41 swinging bucket rotor for 5 h at 4°C. Using an ISCO Model 180 density-gradient fractionator, the sucrose gradient was fractionated and absorbance (*A*) read at 254 nm through the flow cell of an ISCO Model 222 spectrophotometer.

The preceding preparative methods were performed on RNA from normal C3H/HeJ mice (NRNA) and from immunized C3H/HeJ mice.

*RNA treatment of lymphocytes.*—Lymphocytes were obtained from the spleens of normal C3H/HeJ mice. The spleens were teased and cells separated on a Hypaque–Ficoll gradient (Boyum, 1968). The medium used was RPMI 1640 containing 10% heat-inactivated FCS, 20 μg gentamicin/ml (Schering, Kenilworth, N.J.) buffered with HEPES (N-2-hydroxyethylpiperazine-N-2-ethanesulphonic acid, Grand Island Biologicals, Grand Island, N.Y.) at pH 7·4. The lymphocytes were adjusted to a concentration of $2 \times 10^6$/ml and were consistently 97–100% viable as determined by trypan-blue exclusion. Lymphocytes at a concentration of $4 \times 10^6$/2 ml were treated in plastic test tubes (Falcon Plastics, Cherry Hill, N.J.) with 50 μg of DEAE–dextran and a determined amount of RNA (50 μg, 100 μg, *etc.*). The lymphocytes were incubated with the RNA and DEAE–dextran for 30 min at 37°C and 5% $CO_2$. After incubation the cells were washed twice with medium described above and adjusted to a cell concentration of $10^6$/ml for use in the microcytotoxicity assay.

*Microcytotoxicity assay.*—The assay is an adaptation of the Takasugi microcytotoxicity assay (Takasugi and Klein, 1970). A single-cell suspension of 4198V tumour cells, at a concentration of $10^4$/cells per ml, was seeded into wells of 3034 Falcon microcytotoxicity plates (Falcon Plastics, Cherry Hill, N.J.) in 0·01 ml aliquots (100 cells/well). The tumour cells were allowed to attach for 12 h at 37°C in 5% $CO_2$. The wells were washed once with fresh medium and treated lymphocyte suspensions of $10^6$/ml in 0.01 ml aliquots were added to each well. The plates were incubated 40 h, at which time the target cells were washed with serum-free medium, fixed with acetone/alcohol and stained with crystal violet. The cells in the well of each test row were counted and averaged. The percent cytotoxicity was calculated by using the following formula:

$$\%C = \frac{\text{mean of control wells} - \text{mean of test wells}}{\text{mean of control wells}} \times 100$$

*Separation of T and B lymphocytes on nylon- and glass-wool columns.*—Populations of lymphocytes were separated by glass and nylon wool as previously described (Gravely and Kreier, 1976). The numbers of $\theta^+$ cells were determined by incubation of lymphocytes, anti-$\theta$ serum, and complement (Filippi *et al.*, 1976). The percent $\theta^+$ cells was calculated using lymphocytes treated with normal rabbit serum as control. B cells will form rosettes with sensitized complement-coated sheep erythrocytes termed EAC (Bianco *et al.*, 1970). The percent rosettes was calculated on the basis of counting 100 cells after incubation of equal volumes of $10^6$/ml lymphocytes with 1% EAC.

### RESULTS

### *RNA fractionation and sucrose-gradient profiles*

The separation of poly(A)-containing mRNA from total cellular RNA was achieved by the use of oligo(dT)-cellulose. The poly(A)$^+$RNA fraction that elutes with low ionic strength comprised 4–6% of the total material applied to the column. This was confirmed by OD readings each time the column was used.

FIG. 1.—Sucrose-gradient profiles of total cellular RNA (A), poly(A)$^-$ IRNA (B) and poly(A)$^+$ IRNA (C). The RNA (100 $\mu$g) was analysed on linear 5–20% sucrose (w/v) gradients in 0·01M Tris (pH 7·0), 0·05M NaCl and 0·001M EDTA.

Analyses of whole-cell RNA and of the RNA fractions on sucrose gradients are shown in Fig. 1. The profile obtained with whole-cell RNA (A) had 3 peaks: the low-mol-wt (4–6S) RNA which contains transfer RNA, the second peak at about 12–20S which comprises mRNA and smaller ribosomal RNA, and the larger-mol-wt (20–35S) RNA which accounts for larger ribosomal RNA. The profile obtained with poly(A)$^-$RNA (B) closely resembles that found with total cellular RNA. Since poly(A)$^+$RNA makes up such a small amount in comparison to total cell RNA (4–6%), no significant difference should be seen in sucrose-gradient profiles. The sedimentation profile of poly(A)$^+$RNA (C) appeared as one broad peak ranging from 9 to 20S. The broadness of the peak suggests that some contamination with higher-mol-wt ribosomal RNA could be present in the preparation. The profiles obtained with these 3 RNA preparations agree with similar reports using oligo(dt)-cellulose for isolation of poly(A)-tailed mRNA (Green *et al.*, 1976). Unless the sucrose-gradient profiles resembled those shown here, RNA was not used for experimentation.

TABLE I.—*Specificity in the transfer of cytotoxicity*

| Effector cell | IRNA† | Target cell | Target cells‡ per well of test cells ± s.e. | Target cells‡ per well of control cells ± s.e. | Cytotoxicity** |
|---|---|---|---|---|---|
| NL | $IRNA_{(4198V)}$ | 4198V | 30±2 | 52±3 | 42*** |
| $IL_{(4198V)}$ | — | 4198V | 35±3 | 65±2 | 46*** |
| NL | $IRNA_{(4198V)}$ | LM | 50±4 | 52±2 | 4 |
| $IL_{(4198V)}$ | — | LM | 59±3 | 61±2 | 3 |
| NL | $IRNA_{(4198V)}$ | S91 | 47±2 | 53±3 | 11 |
| $IL_{(4198V)}$ | — | S91 | 21±2 | 21±2 | 0 |
| NL | $IRNA_{(LM)}$ | 4198V | 57±3 | 59±2 | 3 |
| $IL_{(LM)}$ | — | 4198V | 61±3 | 65±3 | 6 |
| NL | $IRNA_{(LM)}$ | LM | 30±2 | 54±3 | 44*** |
| $IL_{(LM)}$ | — | LM | 24±2 | 50±2 | 52*** |
| NL | $IRNA_{(S91)}$ | 4198V | 48±2 | 54±3 | 11 |
| $IL_{(S91)}$ | — | 4198V | 20±2 | 19±2 | −5 |
| NL | $IRNA_{(S91)}$ | S91 | 48±2 | 79±3 | 39*** |
| $IL_{(S91)}$ | — | S91 | 29±3 | 42±3 | 31*** |
| NL | — | 4198V | 50±2 | 55±3 | 9 |

* NL, normal lymphocytes; IL, lymphocytes from the spleens of mice immune to cell line given in parentheses.
† RNA extracted from the spleens of mice immune to specific cell lines.
‡ Mean of 18 replicate wells.
** Calculated as described in the text, using lymphocytes treated with normal RNA as control.
*** $P < 0{\cdot}001$.

*Cytotoxicity*

The microcytotoxicity assay was performed using normal lymphocytes treated with 100 μg of total cellular IRNA from 3 different sources. The RNA was extracted from groups of mice immunized separately to the 4198V, LM, and S91 cell lines. As shown in Table I, lymphocytes treated with IRNA extracted from the spleens of 4198V-immune animals transferred 42% tumour-specific cytotoxicity against the 4198V target cell, and 4% and 11% cytotoxicity against the LM and S91 target cells respectively. Likewise, lymphocytes treated with IRNA extracted from LM-immune animals transferred 44% cytotoxicity against the LM cell and 3% against 4198V target cells. Similar results were obtained using IRNA extracted from S91-immune animals. Cytotoxicity was calculated using normal-RNA-treated lymphocytes as control. The viability of cells treated and untreated with RNA remained constant throughout the assay. These results agree with previous work done in our laboratory (Dodd *et al.*, 1973) and others (Kern *et al.*, 1976; Singh *et al.*, 1977).

Poly(A)-tailed mRNA was isolated from total cellular IRNA using oligo(dt)-cellulose chromatography, and normal lymphocytes were treated with doses of poly(A)$^+$IRNA ranging from 1·65 μg to 32·5 μg. As shown in Fig. 2, poly(A)$^+$IRNA transferred optimum activity (50%) when normal lymphocytes were treated with 6·5 μg. Normal lymphocytes were also treated with various doses of poly(A)$^-$IRNA and lower levels of cytotoxicity were transferred against the 4198V target cell. This background level of cytotoxicity obtained with poly(A)$^-$IRNA could be attributed to small amounts of poly(A)$^+$IRNA left in the poly(A)$^-$IRNA fraction. It has been shown after passage through oligo(dT)-cellulose, that poly(A)$^-$RNA contains mRNA lacking a poly(A) tail as well as trace amounts of poly(A)$^+$RNA (Green *et*

FIG. 2.—The dose response curve for the transfer of cytotoxicity to normal lymphocytes by syngeneic poly(A)$^+$ IRNA (●) and poly(A)$^-$ IRNA (○). The control cells were normal lymphocytes treated with various doses of poly (A)$^+$ NRNA and poly (A)$^-$ NRNA. Cytotoxicity calculated as in Table I. Bars represents s.e. (Trivers *et al.*, 1976). The effector: target-cell ratio was 200:1.

FIG. 3. The effect of varying the lymphocyte: target-cell ratio in the transfer of cytotoxicity by 93·5 μg of poly (A)$^-$ IRNA (○) and 6·5 μg of poly (A)$^+$ IRNA (●). The control cells were normal lymphocytes treated with poly(A)$^-$ NRNA and poly(A)$^+$ NRNA. Cytoxicity calculated as in Table I. Bars represent s.e.

*al.*, 1976). Normal lymphocytes treated with various doses of poly(A)$^+$NRNA and poly(A)$^-$NRNA were used as control.

The effect of varying the effector: target-cell ratio from 1:1 to 400:1 is seen in Fig. 3. The optimum dose of poly(A)$^+$IRNA and poly(A)$^-$IRNA was used as determined previously. Maximum activity was observed using a 200:1 effector: target-cell ratio. Increasing the number of lymphocytes beyond this ratio did not increase tumour-specific cytotoxicity.

*Evaluation of glass- and nylon-wool columns*

Splenic lymphocytes were separated on a Hypaque–Ficoll gradient and passed through glass wool to remove macrophages and then through nylon wool to separate and enrich for B- and T-cell populations. For unknown reasons, B lymphocytes stick preferentially to nylon wool (Eisen *et al.*, 1972). Passage of human peripheral-blood lymphocytes through long nylon-wool columns at 37°C was shown to result in a 2–3-fold depletion of complement-receptor lymphocytes (CRL) (Epstein *et al.*, 1971). As shown in Fig. 4, glass- and nylon-wool columns were found to be effective for enriching for CRLs and $\theta^+$ lymphocytes. An average of 55% of the total cell population was recovered after passage through glass wool, and 70% of the glass-wool yield was recovered after passage through nylon wool.

The population eluted from nylon wool was 50% CRLs and 5% $\theta^+$ cells. The non-adherent population was 86% $\theta^+$ cells and 3% CRLs. Even though some cells could not be identified either by a complement receptor or by the $\theta$ antigen, the nylon-wool technique did enrich for the two populations. These percentages agree with those reported previously (Gravely and Kreier, 1976).

FIG. 4.—Evaluation of glass- and nylon-wool columns. Percent $\theta^+$ cells were determined by incubation of lymphocytes, anti-$\theta$ serum and complement, using lymphocytes treated with normal rabbit serum as control. The percent complement-receptor lymphocytes (CRL) was calculated on the basis of 100 cells after incubation of equal volumes of $10^6$ lymphocytes with 1% EAC (sensitized complement-coated sheep erythrocytes). Columns represent the mean of 6 values, bars the range of the values.

*Cytotoxicity with adherent and non-adherent populations of cells*

Treatments with the various RNA components were performed using nylon-wool purified adherent and non-adherent populations of cells in place of unseparated lymphocytes. These were substituted and used in the microcytotoxicity assay. As shown in Table II, no significant levels of cytotoxicity were found, using adherent cells, with any of the IRNA components. In looking at the non-adherent cell population, high levels of cytotoxicity (59%) were found with poly(A)$^+$IRNA. Likewise, 41% cytotoxicity was obtained with treatment with total cellular IRNA and lower levels of 4% with poly(A)$^-$IRNA. These values agreed with those found in treating an unseparated population with these same IRNA fractions.

### DISCUSSION

Poly(A)-tailed mRNA from IRNA was found capable of transferring tumour-specific cytotoxicity. Poly(A)-containing mRNA sediments in sucrose to 9–18S and makes up 4–6% of the total cellular IRNA.

TABLE II.—*Cytotoxicity with adherent and non-adherent populations of normal lymphocytes*

| RNA | RNA/4 × $10^6$ lymphocytes | Effector cell population | Target cells* per well of test cells ± s.e. | Target cells* per well of control cells ± s.e. | % Cytotoxicity† |
|---|---|---|---|---|---|
| Poly(A)$^+$ IRNA | 6·5 μg | non-adherent | 26 ± 2 | 64 ± 2 | 59‡ |
| Poly(A)$^-$ IRNA | 93·5 μg | non-adherent | 51 ± 3 | 53 ± 5 | 4 |
| Total Cell IRNA | 100 μg | non-adherent | 34 ± 2 | 58 ± 2 | 41‡ |
| Poly(A)$^+$ IRNA | 6·5 μg | adherent | 56 ± 4 | 59 ± 3 | 5 |
| Poly(A)$^-$ IRNA | 93·5 μg | adherent | 45 ± 2 | 41 ± 2 | −10 |
| Total Cell IRNA | 100 μg | adherent | 61 ± 3 | 56 ± 3 | −9 |

* Mean of 18 replicate wells.
† Calculated as described in the text, using lymphocytes treated with normal RNA fractions as control.
‡ $P<0·001$.

This agrees with work reported previously in this laboratory that demonstrated fractionation of IRNA on Sephadex G-200, which produced an active component that sedimented from 6 to 12S and made up 4·9% of the total IRNA (Dodd *et al.*, 1973). Several other investigators have shown, using a variety of assay procedures, RNA of this size capable of mediating immune reactions. Fractionation of syngeneic IRNA using preparative sucrose gradients was shown to produce an active anti-tumour fraction (12–16S) which mediated cytotoxicity of murine tumour cells (Kern *et al.*, 1976). An RNA fraction which accelerated skin allograft rejection in rabbits sedimented in sucrose in the 8–18S region (Mannick and Egdahl, 1964). Similarly, an active RNA extracted from human lymph-nodes capable of transferring PPD reactivity to unsensitized cells appeared localized in the 8–12S fraction (Thor and Dray, 1973).

As demonstrated in Table I, RNA extracted from the lymphoid tissue of mice immune to 4198V fibrosarcoma cells transferred specific cytotoxicity for the 4198V target cell and not for the LM or the S91 cell lines. Likewise, RNA from both S91- and LM-immune animal transferred a specific response for each respective target cell. Also, immune lymphocytes isolated from the spleens of each group of mice did not cross-react with target cells of a different origin. The transfer of specific cytotoxicity by syngeneic and xenogeneic IRNA has been shown using other tumour cell line systems (Kern *et al.*, 1974, 1977).

The optimum dosage of poly(A)$^+$IRNA was determined (Fig. 2) to be 6·5 μg. This is the amount of active fraction found in the optimum dosage of total cellular RNA (100 μg). Likewise, 93·5 μg of poly(A)$^-$ IRNA was obtained from 100 μg of total cellular IRNA. A significant difference ($P<0·001$) in percent cytotoxicity was observed between treatment of 6·5 μg of poly(A)$^+$IRNA (50±2%, s.e., Trivers *et al.*, 1976) and 100 μg of total cellular IRNA (42±2%). This suggested that the difference in cytotoxicity was due to further purification of the active RNA fraction, either by removal of substances non-specifically competing with poly(A)$^-$ IRNA, or removal of a specific suppressive fraction of the RNA. Further work in this area is currently under investigation in this laboratory.

The studies performed with adherent and non-adherent cell populations suggested that not only was poly(A)$^+$IRNA the active component of whole-cell IRNA, but also that the tumour-specific cytotoxicity may be due to T-cell-mediated cytotoxicity. Since enrichment of the treated lymphocyte population for T cells did not increase the level of cytotoxicity over that seen with unseparated cell populations, it appears that the maximum level of cytotoxicity for the system has been attained. Cytotoxicity levels of 35–45% observed with tumour-immune lymphocytes in the same assay system substantiate this.

At this point, one can only speculate as to what happens to the RNA once it is taken into the cell. This messenger could enter the cell, attach to the ribosomes, and then begin to translate protein. This protein could change an appropriate cell-surface receptor that commits the cell to a cellular cytotoxic response. The protein could be the light chain of an antibody molecule or a regulatory protein. It is also possible that poly(A)-tailed mRNA could be a template for reverse transcriptase, in which case a DNA copy of this message would be integrated into the cell's genome. At the present time, very little is known about gene regulation in a normal cell, much less what goes on in an immune cell or in a cell given the information from an immune cell.

The active fraction of IRNA responsible for transfer of tumour-specific cytotoxicity has been isolated and further characterized. However, IRNA fractions which mediate other types of immune reactions (*e.g.* blocking antibody and antibody-dependent cell-mediated cytotoxicity) may be present, and further studies on this matter are in progress in this laboratory.

This work was supported, in part, by grants from The National Cancer Institute, National Institutes of Health #Ca-16867, The Ohio Chapter of the American Cancer Society, and from the Graduate School of The Ohio State University, Columbus, Ohio.

### REFERENCES

ADESNIK, M., SALDITT, M., THOMAS, W. & DARNELL, J. (1972) Evidence that all messenger RNA molecules (except histone messenger RNA) contain poly(A) sequences and that the poly(A) has a nuclear function. *J. Mol. Biol.*, **71**, 21.

ALEXANDER, P., DELORME, E. J., HAMILTON, L. D. C. & HALL, J. G. (1967) Effect of nucleic acids from immune lymphocytes on rat sarcomata. *Nature*, **213**, 569.

BELL, C. & DRAY, S. (1973) Lymphoid cells converted by lymphoid RNA extracts *in vitro* and *in vivo* to synthesize allogenic immunoglobulins. *Ann. N.Y. Acad. Sci.*, **207**, 200.

BIANCO, C., PATRICK, R. & NUSSENZWEIG, V. (1970) A population of lymphocytes bearing a membrane receptor for antigen–antibody–complement complexes. *J. Exp. Med.*, **132**, 702.

BILELLO, P., FISHMAN, M. & KOCH, G. (1976) Evidence that immune RNA is messenger RNA. *Cell. Immunol.*, **24**, 58.

BOYUM, A. (1968) Isolation of mononucleic cells and granulocytes from human blood. *Scand. J. Clin. Lab. Invest.*, (suppl.) **97**, 77.

BROWER, P. A., RAMMING, K. P. & DEKERNION, J. B. (1975) Immune cytolysis of human neoplasms mediated by RNA. *Surg. Forum*, **26**, 156.

CLOUDMAN, A. M. (1941) The effect of an extra-chromosomal influence upon transplanted spontaneous tumors in mice. *Science*, **93**, 380.

COHEN, E. P. & PARKS, J. J. (1964) Antibody production by non-immune spleen cells incubated with RNA from immunized mice. *Science*, **144**, 1012.

DECKERS, P. J., WANT, B. S., STUART, P. A. & MANNICK, J. A. (1975) The augmentation of tumor specific immunity with immune RNA. *Transplantation Proc.*, **7**, 259.

DEKERNION, J. B., KERN, D. H., LOUREKOVITCH, H. & PILCH, Y. H. (1974) Intracellular localization of antitumor immune RNA. *Surg. Forum.*, **25**, 123.

DODD, M. C., SCHEETZ, M. E. II & ROSSIO, J. L. (1973) Immunogenic RNA in the immunotherapy of cancer. *Ann. N.Y. Acad. Sci.*, **207**, 454.

EISEN, S., WEDNER, H. J. & PARKER, C. W. (1972) Isolation of pure human peripheral blood T lymphocytes using nylon wool columns. *Immunol. Commun.*, **1**, 571.

EPSTEIN, L. B., CLINE, M. H. & MERRIGAN, T. C. (1971) The interaction of human macrophages and lymphocytes in the PHA-stimulated production of interferon. *J. Clin.Invest.*, **50**, 744.

FILIPPI, J. A., RHEINS, M. S. & NYERGES, C. A. (1976) Antigenic cross-reactivity among rodent brain tissues and stem cells. *Transplantation*, **21**, 124.

GRAVELY, S. M. & KREIER, J. P. (1976) Adoptive transfer of immunity to *Plasmodium berghei* with immune T and B lymphocytes. *Infect. Immun.*, **14**, 184.

GREEN, M., ZEHAVI-WOLLNER, T., GRAVES, P. N., McINNES & PESTKA, S. (1976) Isolation and cell-free translocation of immunoglobulin messenger RNA. *Arch. Biochem. Biophys.*, **172**, 74.

KENNEDY, C. T. C., CATER, D. B. & HARTVEIT, F. (1969) Protection of C3H mice against BP-8 tumor by RNA extracted from lymph nodes and spleens of specifically sensitized mice. *Acta. Pathol. Microbiol. Scand.*, **77**, 796.

KERN, D. H., DROGEMULLER, C. R. & PILCH, Y. H. (1974) Immune cytolysis of rat tumor cells mediated by syngeneic "immune" RNA. *J. Natl. Cancer Inst.*, **52**, 299.

KERN, D. H. & PILCH, Y. H. (1974) Immune cytolysis of murine tumor cells mediated by xenogeneic "immune" RNA. *Int. J. Cancer*, **13**, 679.

KERN, D. H., CHOW, N. & PILCH, Y. H. (1976) Kinetics of synthesis and immunologically active fraction of anti-tumor immune RNA. *Cell. Immunol.*, **24**, 58.

KERN, D. H., DROGEMULLER, C., CHOW, N., HOLLEMAN, D. & PILCH, Y. H. (1977) Specificity of anti-tumor immune reactions mediated by xenogeneic immune RNA. *J. Natl. Cancer Inst.*, **58**, 117.

KUCHLER, R. J. & MERCHANT, D. J. (1956) Propagation of strain L (Earle) cells in agitated fluid suspension cultures. *Proc. Soc. Exp. Biol.*, **92**, 803.

LEE, S. Y., MENDECKI, J. &. BRAWERMAN, G. (1971) A polynucleotide segment rich in adenylic acid in the rapidly labeled RNA component of mouse sarcoma 180 ascites cells. *Proc. Natl. Acad. Sci. U.S.A.*, **68**, 1331.

LIKHITE, V. & SEHON, A. (1972) Cell-mediated tumor allografts immunity: *in vitro* transfer with RNA. *Science*, **175**, 204.

LONDNER, M. V., MORINI, J. C., FONT, M. T. & RABASA, S. L. (1968) RNA-induced immunity against a rat sarcoma. *Experimentia*, **24**, 598.

MACH, B., FAUST, C. & VASSALLI, P. (1973) Purification of 14S messenger RNA of immunoglobulin light chain that codes for a possible light-chain precursor. *Proc. Natl. Acad. Sci. U.S.A.*, **70**, 451.

MANNICK, J. A. & EGDAHL, R. H. (1964) Transfer of heightened immunity to skin homografts by lymphoid RNA. *J. Clin. Invest.*, **43**, 2166.

MITSUHASHI, S., KURASHIGE, S. & KAWAKAMI, M. (1968) Transfer agent of immunity. I. Immune ribonucleic acid which induced antibody formation to Salmonella flagella. *Jap. J. Microbiol.*, **12**, 261.

PAQUE, R. E. & DRAY, S. (1972) Monkey to human transfer of delayed hypersensitivity *in vitro* with RNA extracts. *Cell. Immunol.*, **5**, 30.

PAQUE, R. E. & NEALON, T. (1977) RNA extracts with polyadenylic acid sequences transfer specific sensitivity for a low molecular weight antigen (MW 486). *Cell. Immunol.*, **34**, 279.

PILCH, Y. H., VELTMAN, L. L. & KERN, D. H. (1974) Immune cytolysis of human tumor cells by xenogeneic "immune" RNA: implications for immunotherapy. *Surgery*, **76** (1), 23.

PINCHUCK, P., FISHMAN, M., ADLER, F. & MAURER, P. H. (1968) Antibody formation: initiation in "non-responder" mice by macrophage synthetic polypeptide RNA. *Science*, **160**, 194.

RAMMING, K. P. & PILCH, Y. H. (1970) Mediation of immunity to tumor isografts in mice by heterologous ribonucleic acids. *Science*, **168**, 492.

RIGBY, P. G. (1969) Prolongation of survival of tumor-bearing animals by transfer of "immune" RNA with DEAE dextran. *Nature*, **221**, 968.

SCHLAGER, S. I., PAQUE, R. E. & DRAY, S. (1975) Complete and apparently specific local tumour regression using syngeneic or xenogeneic "tumor immune" RNA extracts. *Cancer Res.*, **35**, 1907.

SINGH, I., TSANG, K. & BLAKEMORE, W. (1977) Effect of xenogeneic immune RNA on normal human lymphocytes against human osteosarcoma cells *in vitro*. *J. Natl. Cancer Inst.*, **58**, 505.

SKINNER, D. G., DEKERNION, J. B., BROWER, P. A., RAMMING, K. P. & PILCH, Y. H. (1976) Advanced renal cell carcinoma: treatment with xenogeneic immune ribonucleic acid and appropriate surgical resection. *J. Urology*, **115**, 246.

TAKASUGI, M. & KLEIN, E. (1970) A microassay for cell-mediated immunity. *Transplantation*, **9**, 219.

THOR, D. E. & DRAY, S. (1973) Transfer of cell-mediated immunity by immune RNA assessed by migration inhibition. *Ann. N.Y. Acad. Sci.*, **207**, 355.

TING, C. C., LAVRIN, D., TAKEMOTO, K. K., TING, R. C. & HERBERMAN, D. (1972) Expression of various tumor-specific antigens in polyoma virus-induced tumors. *Cancer Res.*, **32**, 1.

TRIVERS, G., BRAUNGART, D. & LEONARD, E. J. (1976) Mouse lymphotoxin. *J. Immunol.*, **115**, 130.